# d-Cycloserine enhances durability of social skills training in autism spectrum disorder

**DOI:** 10.1186/s13229-017-0116-1

**Published:** 2017-01-25

**Authors:** Logan K. Wink, Noha F. Minshawi, Rebecca C. Shaffer, Martin H. Plawecki, David J. Posey, Paul S. Horn, Ryan Adams, Ernest V. Pedapati, Tori L. Schaefer, Christopher J. McDougle, Naomi B. Swiezy, Craig A. Erickson

**Affiliations:** 1 Cincinnati Children’s Hospital Medical Center and the University of Cincinnati College of Medicine, 3333 Burnet Avenue MLC 4002, Cincinnati, OH 45229 USA; 2 Christian Sarkine Autism Treatment Center, Riley Hospital for Children at Indiana University Health and the Indiana University School of Medicine Department of Psychiatry, Indianapolis, IN USA; 3 Dr. David Posey M.D., LLC, Indianapolis, IN USA; 4 Lurie Center for Autism, Departments of Psychiatry and Pediatrics, Massachusetts General Hospital and MassGeneral Hospital for Children, Harvard Medical School, Boston, MA USA

**Keywords:** Autism, Autism spectrum disorder, d-Cycloserine, Social skills training

## Abstract

**Background:**

d-Cycloserine (DCS) enhances extinction learning across species, but it has proven challenging to identify consistent benefit of DCS when added to therapeutic interventions. We conducted a placebo-controlled trial of DCS to potentiate social skills training in autism spectrum disorder (ASD) but found substantial improvement in both the DCS and placebo groups at the conclusion of active treatment. Here, we assess the impact of DCS 11 weeks following active treatment to evaluate the impact of DCS on treatment response durability.

**Methods:**

Study participants included 60 outpatient youth with ASD, ages 5–11 years, all with IQ above 70, and significantly impaired social functioning who completed a 10-week active treatment phase during which they received weekly single doses of 50 mg of DCS or placebo administered 30 min prior to group social skills training. Following the 10-week active treatment phase, blinded follow-up assessments occurred at week 11 and week 22. The primary outcome measure for our durability of treatment evaluation was the parent-rated social responsiveness scale (SRS) total raw score at week 22.

**Results:**

Analysis of the SRS total raw score demonstrated significant decrease for the DCS group compared to the placebo group (*p* = 0.042) indicating greater maintenance of treatment effect in the DCS group. DCS was well tolerated, with irritability being the most frequently reported adverse effect in both groups.

**Conclusions:**

The findings of this study suggest that DCS may help youth with ASD to maintain skills gained during sort-term social skills training. Larger-scale studies with longer follow-up will be necessary to further understand the long-term impact of DCS paired with structured social skills training.

**Trial registration:**

ClinicalTrials.gov, NCT01086475

## Background

Social impairment is a defining feature and key predictor of long-term outcome in autism spectrum disorder (ASD) [1, 2]. Social skills training explicitly targets this core deficit and is widely implemented in ASD treatment [2]. Adolescent social skills groups consistently demonstrate immediate improvements in social and communication skills in participants during treatment; however, participants tend to show limited sustained treatment response over longer term follow-up [2–4].

One possible way to improve long-term outcomes of social skills training would be to potentiate the effect of the intervention with adjunctive pharmacotherapy. Recent studies have shown that d-cycloserine (DCS), a partial agonist at the glycine site of the *N*-methyl-d-aspartate (NMDA) receptor, may potentiate response to behavioral therapy [5–9]. DCS appears to potentiate extinction learning, with most significant benefit from intermittent dosing immediately prior to behavioral conditioning in animals or psychotherapy session in humans [10]. The ability of DCS to augment learning appears to be related to NMDA receptor-dependent neural plasticity within the basolateral amygdala [8]. Multiple studies have suggested a role of the glutamate system in the pathophysiology of ASD [11], including rare, disruptive mutations in an NMDA receptor subunit gene [12]. In mouse models of ASD, glutamatergic modulators have been associated with improvement in sociability [12–14], but human studies have been less promising [15–17]. DCS studies in ASD have been primarily short-term monotherapeutic interventions which have not demonstrated consistent, convincing improvements in core features of ASD [18–20]. To date, no studies in ASD have evaluated the combined impact of DCS and therapeutic interventions.

With these facts in mind, our group designed a randomized, double-blind, placebo-controlled trial of low-dose DCS given 30 min prior to weekly peer-mediated group social skills training in youth with ASD. As described by Minshawi et al., at the conclusion of active treatment (week 11), participants in the DCS and placebo groups both demonstrated notable improvement in social functioning, but there was no statistically significant difference between the groups on primary or secondary outcome measures at the end of treatment (primary outcome measure social responsiveness scale (SRS) change score *t* test *p* value = 0.45) [21]. In the current manuscript, we review results from the post-treatment observation phase of the study (week 11 through week 22), during which participants had no further medication or study interventions, but returned for assessment at week 22 with the goal of measuring potential sustained treatment effects of DCS plus social skills training.

## Methods

### Trial design and participants

This study evaluated blinded week 22 durability of treatment data collected following a 10-week randomized, double-blind, placebo-controlled DCS plus peer-mediated social skills group intervention in high functioning youth with ASD completed between August 4, 2009 and January 23, 2014 at Indiana University School of Medicine (IUSM) and Cincinnati Children’s Hospital Medical Center (CCHMC). Please see manuscript by Minshawi et al. for full details describing study design, participants, and statistical analysis of the data collected during and immediately following the 10-week intervention [21]. Both the 10-week intervention and week 22 treatment durability analysis were approved by the institutional review board (IRB) at each participating site (Indiana University Institutional Review Board and Cincinnati Children’s Hospital Medical Center Institutional Review Board). Guardians of all participants provided written informed consent prior to study enrollment. Assent was obtained from enrolled youth when possible.

Study participants were youth ages 5–11 years recruited from outpatient psychiatric clinics at both sites and via IRB approved advertising. Participants were diagnosed with autistic disorder, Asperger’s disorder, or pervasive developmental disorder, not otherwise specified (PDD-NOS) by clinical assessment based on the Diagnostic and Statistical Manual of Mental Disorders, Fourth Edition Text revision (DSM-IV-TR) diagnostic criteria [22], and corroborated by administration of the Autism Diagnostic Observation Schedule (ADOS) [23] and the Autism Diagnostic Interview-Revised (ADI-R) [24]. Due to the cognitive and verbal demands of the social skills intervention, subjects were required to have an Intellectual Quotient (IQ) above 70 as measured by the Stanford-Binet Fifth Edition (SB-V) [25] and a Vineland Adaptive Behavior Scale Second Edition (VABS-II) [26] communication standard score above 70. At baseline, all subjects demonstrated significant social impairment with T-scores of 60 or greater on the SRS [27] and scores of 70% or less on both the parent questionnaire and child assessment portions of the Triad Social Skills Assessment (TSSA) [28].

Concurrent psychotropic medication use was permitted, though all subjects were required to remain on stable doses throughout the treatment intervention and through week 22 durability of treatment effect analysis. Participants were excluded if treated with more than two psychotropic medications or known glutamatergic modulators such as riluzole, memantine, acamprosate, topiramate, or amantadine among others. Participants enrolled in psychosocial interventions independent from the study protocol were required to have stable regimens throughout the study. Participants were excluded from the study if they were participating in concurrent group social skills training programs.

For the 10-week intervention phase of the study, participants were enrolled in a series of 17 social skills groups (13 at IUSM and 4 at CCHMC), each containing four children with ASD and two typically developing, age-matched peer models. Peer models were determined to be free of psychiatric symptoms or developmental disability via the Child Symptom Inventory-4 [29] and a medical and psychiatric interview completed by a study physician. Subjects with ASD were randomized to either DCS or placebo in a 1:1 ratio by computer-generated randomization list accessible only by the investigational pharmacist. A dose of 50 mg DCS or placebo was administered 30 min prior to 10 weekly, 2-h sessions of manualized social skills training. Social skills intervention followed a curriculum utilizing ABA-based techniques designed to teach skills including greetings, understanding emotions, creative play, and social conversations [30]. Social skills groups were divided by age (5–7 years or 8–11 years), and minor modifications were made to the group curriculum to accommodate the different age ranges. Groups were instructed by masters or doctorate level clinicians with specific expertise and experience working with youth with ASD.

Following the 10-week intervention phase, participants received no ongoing study-related therapeutic intervention or treatment with study drug. Participants were asked to return at week 11 and week 22 for blinded assessment. Participants, caregivers, and investigators remained blind to study group-assignment until after all week 22 follow-up visits were completed, all data was recorded in a RedCap database, quality checks were completed, and the data set was locked.

**Fig. 1:**
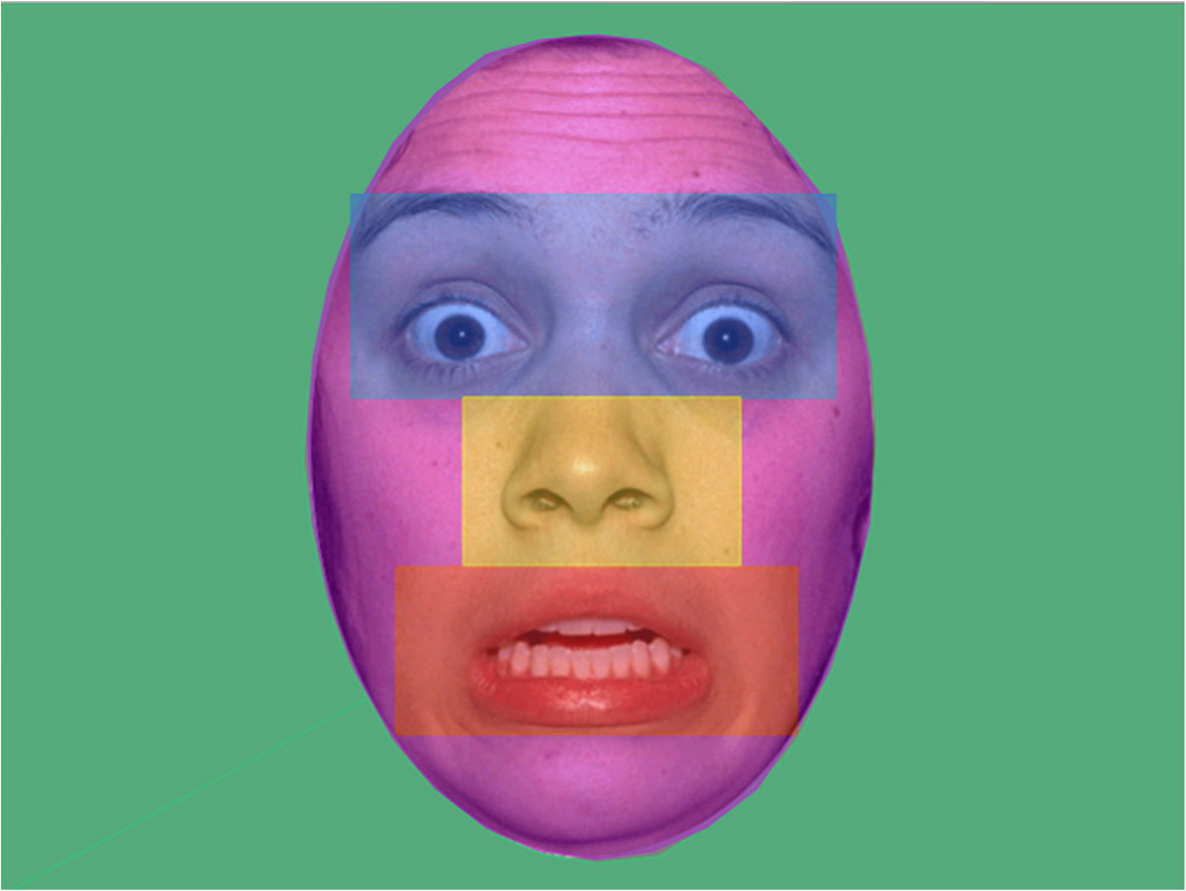
Face image from the NimStim Face Stimuli Set with highlighted areas of interest. The individual pictured in this figure was not a study participant

Finally, we incorporated a pilot eye-tracking paradigm of gaze preference employing a Tobii T120 Infrared Eye Tracker integrated with a 17-inch thin film transistor monitor controlled with Tobii Studio software (Version 3.0). Eye-tracking assessment was completed at week 11 and week 22 in a subset of participants beginning in year 3 of the project. In this pilot assessment, participants viewed 60 colored photographs of adult human faces from the NimStim Face Stimuli Set [31] and percent time looking at the eye, nose, mouth, or whole face region was calculated (Fig. 1). Time spent viewing the eye region and face as a whole implied greater social interest in comparison to time spent looking at other facial regions [32–34].

### Outcome assessments and statistical analysis

Demographic data were collected at screen, prior to randomization to study group. Monitoring for adverse events was completed by a study physician at all visits via discussion of new symptoms, recent doctor visits, and current medication review. The primary outcome measure at week 22 (and at week 11) was the parent-rated SRS total raw score. Exploratory outcome measures at week 22 were the SRS subscale component scores. The SRS total raw score ranges from 0 to 195, with higher scores indicating more significant social impairment [27].

Demographic data describing the week 22 completers in the DCS and placebo groups (age, gender, ASD subtype, and concomitant medications), as well as reported adverse events/frequencies were compared using Student’s *t* tests for continuous variables and Fisher’s exact tests for categorical variables. Wilcoxon rank sum tests were completed to validate results of the Student’s *t* tests.

To assess potential impact of participants lost to follow-up, we compared using Wilcoxon rank sum tests the week 22 completers versus those who dropped out before week 11 on the SRS total raw score at baseline. We also compared these completers to those who dropped out before week 22 on the change in SRS total raw score from baseline to week 11.

To examine the differences between the groups on SRS total raw scores from week 11 to week 22, we employed a robust linear model based on M-estimation, specifically the bisquare weight function (or biweight), which is the default method in the SAS ® statistical software package (SAS ® version 9.3, SAS Institute Inc., Cary, NC). A robust linear model was used because preliminary analyses indicated that the data contained some outliers. Rather than omitting these points (i.e., setting their weight to zero) and fitting parameters using least squares, we preferred to downweight the outliers based on their distance from the bulk of the data. The biweight was used because it provides smoothly changing weights to all of the observations, including the outliers [35]. In our model, the response was the SRS total raw score at week 22 and the independent variable was treatment group (DCS or placebo). The weightings account for the SRS total raw score at week 11 as well as the covariates gender, age group (5–7 versus 8–11 years), and ASD diagnosis (autistic disorder versus Asperger’s disorder and PDD-NOS combined). Since this model is predicting week 22 SRS total raw scores while controlling for week 11 SRS total raw scores with the weightings, this model predicts relative change in this measure from week 11 to week 22. The weighted estimates are similar to means, but are more resistant to the adverse effects of outliers, and refer to the effect plus the value of the biweight estimator at week 22. The biweight estimator is a weighted average of the week 22 results; adding this value to the treatment effects allows the estimates to be comparable to raw values.

The effect sizes resulting from the robust linear model described above were calculated by dividing the magnitude of treatment effect by the robust measure of scale produced by the model above. In the spirit of Cohen’s *d*, which is based on means and the standard deviation, we will refer to this effect size as *d*_biw_, to indicate that this effect size is based on robust biweight estimators. Similar versions of the effect size based on different robust models have been discussed by Wilcox and Tian [36]. To correct for multiple exploratory post hoc comparisons, false discovery rate (FDR) procedures were utilized for the secondary SRS subscale analyses.

For the pilot eye-tracking data, a repeated measures linear mixed model was conducted where the response was the percent fixation time at a particular area of interest (SAS ® version 9.3). The continuous covariate was the baseline percent fixation time and the categorical independent variables were treatment group and week 11 or week 22 as well as their interaction term. The “week” term was the repeated measure within subjects (which is the random effect). Other covariates included the face identifier, expression, mouth (open or closed), age group, gender, and ASD subtype. The least squares means were derived for each treatment by week combination. The contrast comparing the treatment change between the two weeks of interest was then derived.

## Results

### Review of week 11 findings [21]

In brief, the 10-week intervention phase of the study, described in detail by Minshawi et al., enrolled 68 children with ASD with no statistically significant treatment group differences in demographics, clinical factors, concomitant medications, therapeutic interventions, or SRS total raw score at baseline [21]. Mean SRS total raw score decreased during the intervention phase in both groups. At week 11, the SRS change scores from baseline demonstrated no statistically significant difference attributable to DCS treatment. DCS was well tolerated, with irritability being the most frequently reported adverse effect in both groups. There was no statistically significant difference in number of reported adverse events between groups. A supplementary figure providing a comprehensive overview of the mean SRS total raw scores across all study phases, including both the initial study treatment phase (week 11 endpoint) and the long-term follow-up (week 22 endpoint) was included as a supplementary file for reference (please see Additional File 1).

### Week 22 findings

**Fig. 2:**
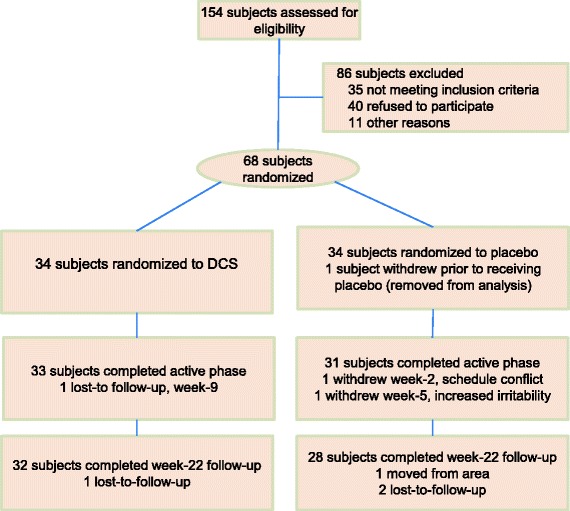
Consort diagram

Sixty participants completed week 22 (four subjects were lost during active treatment phase and four lost during follow-up period, consort diagram, Fig. 2). At week 22, there were no statistically significant differences between treatment groups in age, gender, diagnostic subtype, or concomitant medications (Table 1). In our examination of the impact of participants lost to follow-up, we found no statistically significant difference between the week 22 completers and those who were lost to follow-up prior to week 11 on the SRS total raw score at baseline (Wilcoxon Rank Sum *p* value = 0.90). Furthermore, we found no statistically significant difference when we compared these completers to those who dropped out before week 22 on the change in SRS total raw score from baseline to week 11 (Wilcoxon rank sum *p* value = 0.91).

**Table 1:** Demographic characteristics of week-22 completers

	DCS^a^ (*n* = 32)	Placebo (*n* = 28)	*p* value
Characteristics			
Age (years), mean (SD^a^)	8.47 (1.83)	8.07 (1.53)	0.37
Sex, *n* (%) male	26 (81.35)	23 (82.14)	1.00
Diagnosis, *n* (%) autistic disorder	12 (37.50)	12 (52.86)	0.79
Concomitant medications, *n* (%)			
Antipsychotics	6 (18.75)	4 (14.29)	0.74
Alpha-2 agonists	6 (18.75)	6 (21.43)	1.00
Stimulants	11 (34.38)	7 (25.00)	0.57
Antidepressants	3 (9.38)	3 (10.72)	1.00
Atomoxetine	1 (3.13)	1 (3.57)	1.00
Any concomitant medication	19 (59.39)	17 (60.71)	1.00
Mean total concomitant medications	0.84	0.75	0.64

*SD* standard deviation

^a^ *DCS* d-Cycloserine

The robust linear model employed in this analysis to examine the difference between DCS and placebo groups on SRS total raw scores from week 11 to week 22 demonstrates that the DCS group decreased significantly compared to the placebo group (DCS mean estimate = 85.1 (SE 7.3), placebo mean estimate = 91.5 (SE 7.6), DCS-placebo estimate (treatment effect) = −6.4 (SE 3.1), *p* = 0.042, *d*_biw_ = 0.69). This suggests that DCS increased durability of social skills training gains at week 22 in those children treated with active drug. These differences account for week 11 SRS scores as well as gender, age, and diagnosis because of the robust linear model used in analysis. Effect size is in the moderate to large range, indicating that the observed change was meaningful [37]. The size of the effect is apparent in that the treatment effect (the difference between the DCS estimated mean and the placebo estimated mean) is only slightly smaller than the SEs of each of the estimated means.

In the secondary analysis of the SRS subscales, the social cognition subscale showed the greatest between groups difference (*p* = 0.003, *d*_biw_ = 0.82). When we corrected for multiple exploratory comparisons utilizing FDR procedures, this effect remained significant. There were no statistically significant differences between groups on the social awareness, social communication, social motivation, and autistic mannerism subscales.

Finally, the pilot eye-tracking measure was completed in 38 subjects, 21 in the DCS group and 17 in the placebo group. There was no statistically significant difference identified between these groups in age, sex, diagnostic subtype, or concomitant medications. Results demonstrated that the DCS treatment group had decreased percent time looking at the nose, but increased percent time looking at the face as a whole (*p* < 0.0001) when compared to the placebo group. There was no difference in percent time spent viewing the eye or mouth regions between groups. As the social skills curriculum employed in this study directly taught and reinforced eye gaze to the face and eyes region, this data suggests that the DCS treated group was potentially more successful at gaining this skill and was therefore more socially interested in viewing faces in the eye-tracking paradigm at week 22 as a result of receiving active drug.

## Discussion

To our knowledge, this study combing social skills therapeutic intervention with medication is the first of its kind to be completed in ASD research. The week 11 results reported by Minshawi et al. demonstrating decreased SRS total scores in both treatment groups but not between group treatment effects align with previous reports of substantial immediate impact of social skills training in youth with ASD [2]. In this follow-up study, we show enhanced durability of treatment response in those subjects who received weekly DCS, a novel finding considering previous studies indicating lack of treatment durability following social skills intervention in ASD [4]. In this analysis, DCS appears to support maintenance of social skills gains made during short-term group therapy compared to placebo. This improved maintenance of effect was measured both by parent-reported increased social cognition and by our pilot eye-tracking paradigm. Furthermore, this medication’s long track record of safe use in children, limited adverse effects in this study, and demonstrated impact with intermittent dosing in other disorders suggests significant potential for future treatment development of DCS in ASD.

There are several limitations of our trial design that must be considered when interpreting the results of this study. First, we utilized a novel, peer-mediated manualized social skills training curriculum for this study [30]. Although the curriculum is based on validated ABA-based techniques, the curriculum itself has not been validated outside of this study. Second, the social skills training design limited this intervention to a selective group of children with relatively high communication and adaptive functioning, limiting the inferences that can be made regarding intermittent DCS dosing to support learning in youth with ASD more generally. Third, the SRS was designed as an assessment tool rather than as an outcome measure, the subscales were derived via expert consensus and may not reflect a true underlying construct, and the large range of potential scores present a challenge in interpreting the exact meaning of a score change [27, 38]. However, we made the choice to employ this measure as our primary outcome as this measure was most in line with the core social impairments we hoped to address via our study intervention, and has also been used in other social skills training studies in youth with ASD [39]. Fourth, our eye-tracking paradigm was piloted only in a subgroup of participants in this study, limiting the impact of these results. Future studies could continue to explore the use of eye tracking as a potential objective measure of social interest by including all study participants in the trial design. Last, the specific mechanisms underlying the longer term benefits of DCS, such as reduction of social anxiety during treatment or improvements in learning and memory during therapy, remain to be established. Despite these limitations, the prospect of enhancing the sustained benefit of social skill training interventions with pharmacotherapy is novel and exciting. Given that social skills training is a primary intervention for ASD but one with limited long-term benefits, the added impact of DCS could significantly improve the long-term social functioning of children with ASD. Replication of the observed effects would require a larger study that is explicitly focused on sustained benefit. Additional work is also needed to determine the mechanisms underlying the benefit of DCS added to social skills training.

## Conclusions

Adjunctive DCS significantly increased the sustained benefit from short-term social skills intervention 3 months after treatment cessation. This is of importance considering the body of literature suggesting limited durability of therapeutic interventions targeting core features of ASD. Additionally, the safety and time limited nature of this drug treatment, as demonstrated by the limited adverse effects reported by study participants, indicates that DCS may be a safe and effective strategy to enhance the durability of therapy impact in youth with ASD. This finding holds significant potential in ASD where there are no approved treatments for the core social skills deficits associated with the disorder.

## Abbreviations

ADI-R: Autism Diagnostic Interview-Revised
ADOS: Autism Diagnostic Observation Schedule
ASD: Autism spectrum disorder
CCHMC: Cincinnati Children’s Hospital Medical Center
DCS: d-Cycloserine
DSM-IV-TR: Diagnostic and Statistical Manual of Mental Disorders, Fourth Edition Text revision
IQ: Intellectual quotient
IRB: Institutional review board
IUSM: Indiana University School of Medicine
NMDA: *N*-methyl-d-aspartate
PDD-NOS: Pervasive developmental disorder, not otherwise specified
SB-V: Stanford-Binet Fifth Edition
SRS: Social responsiveness scale
TSSA: Triad Social Skills Assessment
VABS-II: Vineland Adaptive Behavior Scale Second Edition
